# A novel caloric restriction mediator

**DOI:** 10.18632/aging.101311

**Published:** 2017-10-25

**Authors:** Masaki Kobayashi, Yoshikazu Higami

**Affiliations:** Laboratory of Molecular Pathology and Metabolic Disease, Faculty of Pharmaceutical Sciences, Tokyo University of Science, 2641 Yamazaki, Noda, Chiba 278-8510, Japan

**Keywords:** caloric restriction (CR), sterol regulatory element binding protein-1c (Srebp-1c)

Caloric restriction (CR) extends lifespan and suppresses age-associated pathophysiology in various animal models [1]. However, the exact mechanisms underpinning these effects are still debated. White adipose tissue (WAT) is the main tissue for energy storage in the form of triglycerides. Moreover, it is accepted that WAT contributes to systemic metabolic dysfunctions including insulin resistance and cardio- and cerebro-vascular diseases. Thus, the characteristics of WAT appear to influence age-associated patho-physiology and lifespan [2]. In fact, it has been reported that various WAT-specific genetically modified models exhibit alterations in lifespan (references are presented in [3] and [4]).

CR animals share many characteristics with long living dwarf mice with suppression of growth hormone/insu-lin-like growth factor (GH/IGF-1) signaling. However, CR further extends the lifespan of these mice, suggesting that the beneficial effects of CR are not only dependent on GH/IGF-1 signaling. Therefore, to identify genes altered by CR in GH/IGF-1-dependent or -independent manners, we previously compared gene expression profiles of WAT between CR rats and transgenic dwarf rats, bearing an antisense GH transgene. This analysis showed that CR induced expression of sterol regulatory element binding protein-1c (Srebp-1c) in a GH/IGF-1-independent manner [3]. Srebp-1c is one of the SREBP isoforms, master transcriptional regulators of lipid metabolism. In agreement with this function, CR also upregulated the expression of genes involved in fatty acid (FA) biosynthesis in a GH/IGF-1-independent manner [3]. In this previous study, we proposed that Srebp-1c-induced activation of FA biosynthesis is one of the major mechanisms by which CR changes metabolism in WAT [3]. Thus, we concluded that activation of *de novo* FA biosynthesis via Srebp-1c in WAT may be pivotal for the beneficial effects of CR.

To investigate the involvement of Srebp-1c in CR effects in detail, we used Srebp-1c wild-type (WT) and knockout mice (KO) fed *ad libitum* (AL) or subjected to CR. First, we confirmed CR-associated induction of Srebp-1c mRNA in WAT of both fed and fasted mice. Moreover, CR upregulated proteins implicated in FA biosynthesis, including fatty acid synthase (Fasn), acetyl-CoA carboxylase (Acc), ATP citrate lyase (Acly), and malic enzyme-1 (Me-1), in WAT of WT. An increase of these proteins was not observed in KO [4]. These results support that CR activates *de novo* FA biosynthesis in WAT via Srebp-1c.

Various reports have demonstrated that CR enhances mitochondrial biogenesis in several tissues [5]. We focused on the relationship between mitochondrial biogenesis and Srebp-1c. Unlike previous reports, our results showed that CR did not increase three proteins implicated in mitochondrial biogenesis, translocase of outer mitochondrial membranes 20 kDa (Tom20), cytochrome c oxidase subunit 4 (Cox4), and sirtuin 3 (Sirt3), in the liver, kidneys, skeletal muscle, or heart. However, in WAT of WT, CR significantly induced expression of these proteins. In addition, CR enhanced citrate synthase activity, a rate-limiting enzyme of the tricarboxylic acid cycle, and increased mitochondrial DNA content. However, the CR-associated changes were attenuated in KO [4]. Peroxisome proliferator-activated receptor γ coactivator-1α (Pgc-1α) is known to play a critical role in CR-associated mitochondrial biogenesis. In our analysis, CR induced expression of Pgc-1α in WAT of WT, but not in KO [4]. Moreover, we demonstrated that Srebp-1c occupies the Pgc-1α promoter region, where two sterol regulatory elements (predicted as Srebp-1c-binding sites) are located, using chromatin immunoprecipitation assays [4]. These results suggest that CR induces transcription of Pgc-1α through Srebp-1c binding to its promoter, thereby enhancing mitochondrial biogenesis in WAT.

Generally, CR suppresses oxidative stress [6], and mitochondrial functions are closely related to oxidative stress. Therefore, we evaluated two biomarkers of oxidative stress, activity of aconitase, a mitochondrial enzyme vulnerable to oxidative stress, and the ratio of oxidized glutathione to reduced glutathione (GSSG/GSH). Consequently, CR significantly increased aconitase activity and decreased the GSSG/GSH ratio in WAT of WT, but not in KO. In contrast, the CR-associated reduction was not observed in other tissues [4]. Collectively, these results suggest that Srebp-1c may be required for CR-associated activation of mitochondrial biogenesis and suppression of oxidative stress, specifically in WAT.

**Figure 1:**
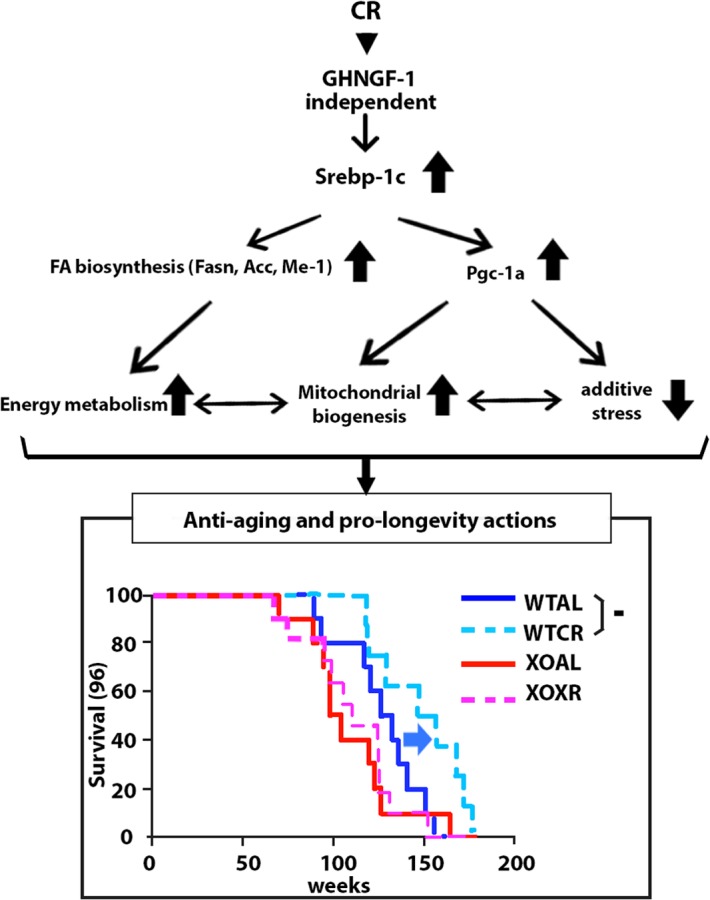
Diagram demonstrating the proposed novel molecular mechanism of beneficial CR-associated metabolic changes in WAT and longevity effects, and survival curves of WT and KO fed AL (WTAL and KOAL) and subjected to CR (WTCR and KOCR) groups.

In the present study, we revealed for the first time that Srebp-1c is involved in CR effects on FA biosynthesis, mitochondrial biogenesis, and oxidative stress in WAT, but not in other tissues. These findings support our previous hypothesis that induction of FA biosynthesis and mitochondrial biogenesis by CR may represent a shift of the substrate type used for whole body energy from carbohydrates to lipids [7]. Additionally, suppression of oxidative stress in WAT may contribute to alterations of adipokine profiles and anti-inflam-matory responses observed in CR animals. Finally, we confirmed that CR extends the lifespan of WT, but not KO [4]. Therefore, Srebp-1c orchestrates the CR-associated metabolic remodeling through effects on lipid metabolism, mitochondrial biogenesis and oxidative stress in WAT in a GH/IGF-1-independent manner, resulting in the extension of lifespan (Figure 1).
